# Arctic corridors and northern voices project: Methods for community-based participatory mapping for low impact shipping corridors in Arctic Canada

**DOI:** 10.1016/j.mex.2020.101064

**Published:** 2020-09-12

**Authors:** Jackie Dawson, Natalie Ann Carter, Nicolien van Luijk, Melissa Weber, Alison Cook

**Affiliations:** Department of Geography, Environment and Geomatics, University of Ottawa, Canada

**Keywords:** Community-based research, Participatory mapping, Youth engagement, Arctic, Low impact shipping corridors, Local and Inuit knowledge, ACNV, Arctic Corridors and Northern Voices, ESPG, Environment Society and Policy Group, HTA, Hunters' and Trappers' Associations

## Abstract

Documenting Inuit and local knowledge is critical to its consideration within policy discussions around Arctic shipping; especially considering the rapid increase in ship traffic due to reductions in sea ice and climate change. We present our unique community-based research approach which incorporated youth training, participatory mapping, qualitative focus group discussions, and verification exercises to document Inuit communities’ perspectives in Arctic Canada about Low Impact Shipping Corridors. These qualitative activities provided appropriate context and understanding around community-created maps, community-identified opportunities, concerns, and recommendations, and the policy relevance and feasibility of recommendations posed. Three activity phases were employed; 1) before engaging in in-community research, 2) during in-community research, and 3) after completing in-community research. Spatial and non-spatial data were analyzed using ArcGIS® and NVivo software, respectively. These methods and observations can inform future research initiatives, particularly transdisciplinary teams, including those involving southern-based (early career) researchers, working in Inuit Nunangat.

• Methods presented here ensured that scientific processes and outputs were robust and rigorous and research was conducted in a respectful, reciprocal manner.
• Only through the collaborative efforts of a transdisciplinary team could scientific rigour be attained and respect be afforded.
• The approach can be easily applied to document community members’ perspectives on local priorities.

## Graphical abstract

**Figure:**
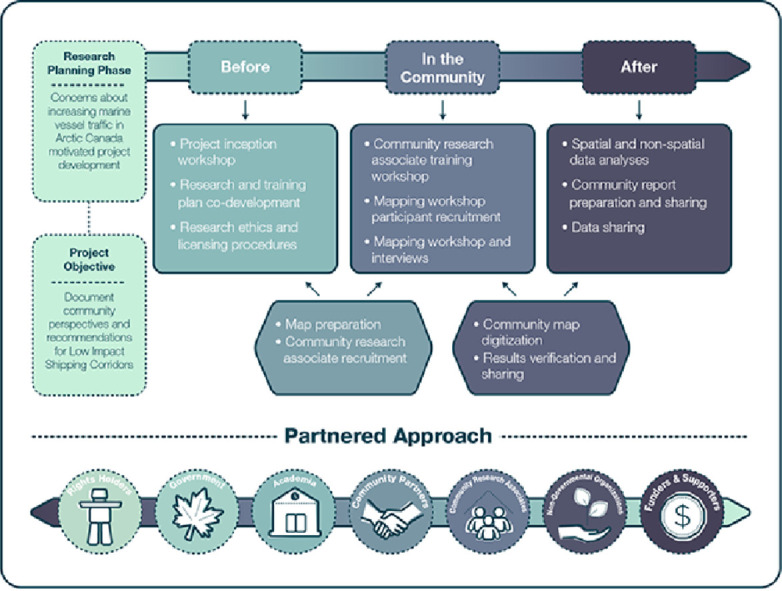

Specifications table

**Table:** 

Subject Area	Environmental Science
More specific subject area	*Arctic shipping and climate change*
Method name	*Community-based research and participatory mapping*
Name and reference of original method	[17]*. Living proof: The essential data-collection guide for Indigenous use-and-occupancy map surveys.* Vancouver: Ecotrust Canada.

## Method details

In this paper we present the details, design, and methodological approach used in the Arctic Corridors and Northern Voices (ACNV) research project. The ACNV project was established in 2014 in direct response to the vital need to consider Inuit and local knowledge within policy discussions around Arctic shipping, especially considering the rapid increase in ship traffic due to reductions in sea ice and climate change [7,^13^,^14^,16]. The aim of the project was to generate an inventory and foundation of scientific and Inuit knowledge about shipping impacts and culturally significant marine areas and to map this knowledge spatially for consideration within the development and implementation of ‘Low Impact Shipping Corridors” across Arctic Canada (formerly ‘Northern Marine Transportation Corridors’; see [3] also www.arcticcorridors.ca). ‘Low Impact Shipping Corridors’ is a Government of Canada-led initiative with the goal of developing low-impact marine transportation corridors in the Arctic Ocean to encourage marine shipping and transportation traffic to use routes that pose less risk and to minimize the impact on communities and the environment [13,14]. Despite recommendations made by The Arctic Council over ten years ago that corridors should consider Inuit and northern (i.e., non-Inuit long-term residents) perspectives and culturally significant marine areas in their development, this has yet to be fully realized (AMSA 2009). Through the ACNV project we endeavoured to respond to this clear information gap and to document those perspectives.

The ACNV project utilized a collaborative methodological approach wherein southern-based university researchers involved regional and national decision makers and partnered with northern-based Inuit and northern community members during all stages of the research process. This approach was designed to actively facilitate cross-cultural (northern- and southern-based) knowledge and skills exchange and to facilitate a “co-learning” experience, which is known to elicit stronger research outcomes (see [1]). A community-based research approach was employed where the goals involved co-generating research that is relevant at a local scale, and that enhances community-involvement and local research capacity [2,11]. Within this community-based research approach we also adopted participatory mapping methods. Participatory mapping involves the creation of spatial maps of certain phenomena that represent what the community perceives to be important to them, including natural and socio-cultural features. Both community-based research and participatory mapping provide opportunities to document Inuit and local knowledge, and have been proven to play a highly effect role in policy and decision-making processes ([2]; International Fund for Agricultural Development [9], 2009; [11]). In conjunction with these methods, we also conducted formal and informal focus group discussions and semi-structured interviews with subject matter experts, relevant federal, regional, and local decision makers, and community members. These qualitative activities were used to provide appropriate context and understanding around community-created maps, community-identified opportunities, concerns, and recommendations, and the policy relevance and feasibility of recommendations posed.

**Fig. 1:**
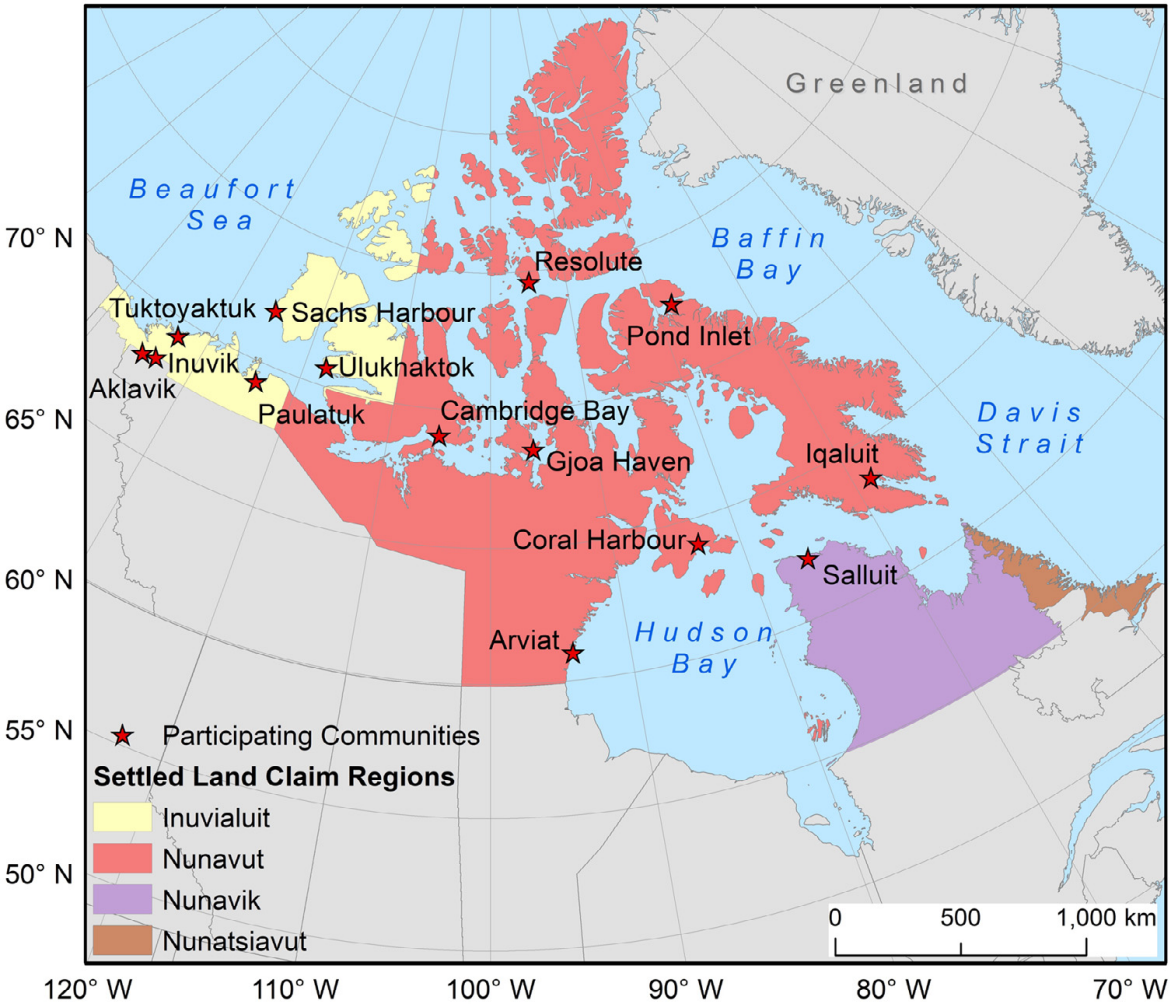
Participating Communities in Inuit Nunangat (Inuit homeland in Arctic Canada).

Fourteen communities from three different regions in Arctic Canada participated in the ACNV project including, six (all) from the Inuvialuit Settlement Region; Aklavik, Inuvik, Paulatuk, Sachs Harbour, Tuktoyaktuk, and Ulukhaktok; seven from Nunavut (which consists of three different regions – Kivalliq, Kitikmeot, Qikiqtaaluk): Arviat, Iqaluktuuttiaq (Cambridge Bay), Salliq (Coral Harbour), Uqsuqtuuq (Gjoa Haven), Iqaluit, Mittimatalik (Pond Inlet), and Qausuittuq (Resolute); and one from Nunavik: Salluit (see Fig. 1). All of these communities are impacted by climate change, sea ice reduction, and increased shipping [15].

An important aspect of our approach to participatory mapping in each community was involvement of local youth as co-researchers, hereafter referred to as ‘community research associates’. During the course of the research project, 59 Inuit and Northern youth (aged 15-45) were trained in mapping and data collection methods. These community research associates then recruited 133 expert knowledge holders who participated in the community mapping workshops, focus group discussions and interviews which were co-facilitated by southern-based researchers and community research associates. Overall, southern-based team members spent on average a total of 22 days in each case study community including five days for relationship building, project mobilization and planning, 12 days for community research associate training, community mapping workshops, and wrap-up, and five days during a second trip for results verification and sharing.

In this paper, we provide an in-depth overview of the methods involved in this research project, which are organized into three major phases; (1) before engaging in in-community research activities, (2) during in-community research activities, and (3) after the in-community research activities were completed (see graphical abstract for a concise overview). Within these phases we discuss the facilitation of community research associate training workshops, community mapping workshops, qualitative focus group discussions and interviews, and results verification and sharing exercises. The three research stages each played an integral part in our methodological approach, and while, for the sake of clarity in this paper, the sections are separated, our approach involved an interactive back-and-forth process to ensure research rigour.

## Overview of three-phased methodological approach

In the ‘Before’ section of this paper, we describe how we co-developed our research objectives and methods, selected and approached (potential) partner communities, planned community mapping workshops, prepared our mapping methods, and identified community research associates. We also briefly describe the research ethics and licensing procedures completed during this phase.

In the ‘In the Communities’ section of the paper we discuss in detail our data collection (knowledge documentation) methods including the development of co-learning and training workshops with community research associates, facilitation of community mapping workshops and interviews, digitization of community mapping results and the facilitation of community events where results were shared and verified.

In the ‘After’ section of this paper we provide insight into how we: edited the mapping data post-community verification, how we analysed our interview and community workshop data and also how we integrated the spatial (mapping) and non-spatial (focus group and interview) data to enhance research rigour and prepare for the publication of community reports and academic manuscripts. We also discuss the process of sharing results and data with participant communities and other partners (e.g., non-governmental organizations), as well as rights holders (e.g., Inuit organizations) and stakeholders (e.g., Government of Canada agencies), including returning proprietary local knowledge to communities for ownership, stewardship, and archiving.

## Before in-community research activities

### Project inception workshop and co-development of research objectives and tools

The ACNV project was conceptualized during a multi-stakeholder workshop about Arctic shipping and Low Impact Shipping Corridors that was jointly organized by the University of Ottawa, the Canadian Coast Guard (a Government of Canada agency), and Oceans North Canada (a non-governmental organization; now Oceans North). The workshop was held at the 2015 ArcticNet annual science conference in Ottawa, Canada and involved over 40 participants including rights holders (Inuit); academics; government, non-governmental and Inuit organization representatives; and industry stakeholders (see [8]). The ACNV project aim, objectives, and plan were co-created by a selection of stakeholders and rights holders who attended this workshop or who were recommended by individuals in attendance (and contributed post workshop). During the workshop, participants identified the need to document Inuit knowledge about shipping impacts and culturally significant marine areas, as well as recommendations for Low Impact Shipping Corridors governance. Participants proposed mapping this knowledge spatially and temporally (by season) for consideration within the development and implementation of the Low Impact Shipping Corridors. They also recommended the involvement of community partners and youth community research associates to enhance community engagement, scientific rigour, and local research capacity. Following the workshop, a selection of workshop participants drafted and submitted an application to a competitive research grants competition and were ultimately successful. During this effort the team also iteratively co-developed draft questions for community mapping workshops and interviews that would achieve intended project objectives to identify culturally significant marine areas and local concerns and recommendations for marine vessel management through a corridors approach.

This co-creation of the research project aim, objectives, and questions helped to ensure that the approach was locally relevant and that partnerships and agreement on priorities were established from the beginning. We credit this approach as having played a large part in the success of the project. Between 2014 and 2020, 15 competitive science grants and sole source contracts were obtained to support the study, which totalled over CAD1.5 million (USD1.1 million at the time of submission).

### Co-development of project and mapping methods

#### Selection of community case studies and partners

The first three communities that participated in the ACNV project were chosen because of previous research relationships and connections between the southern-based university researchers and the northern-based Inuit communities who had expressed concerns about increasing marine vessel traffic. As funding became increasing available an additional 11 community case studies were added and these were selected based on several factors including; invitations received by the research team from community organizations who had heard about the project, geographical location, and volume of marine vessel traffic nearby. Through existing community relationships and research networks, southern-based team members contacted community organizations located in the communities in which the team wanted to work such as, Hunters' and Trappers' Associations (HTA), Hamlet Councils, Village Offices or Community Corporations; Ikaarvik Barriers:Bridges Program, and Arviani Aqqiumavvik Society. These contacts were made by southern-based team members via introductory emails and letters, follow-up phone calls, provision of example community reports, and a 5-minute video to introduce the project to potential partners (see https://www.youtube.com/watch?time_continue=1&v=q8PPqilwGUk&feature=emb_logo). Introductory emails and letters (translated) in non-technical language outlined; (1) why the research was likely to be important and relevant to the community, (2) who would work on this research from inside and outside the community, (3) how the community organization would ideally support the research (e.g., identify community research associates and research participants); and (4) how research results would be shared in the community (e.g., radio call-in show, open house).

Pre-Workshop Planning

It was decided by the project team that all community mapping workshops would be co-facilitated with local youth (community research associates) in each community. This approach was recommended by project partners and it proved to be highly successful in building local research capacity, ensuring appropriate recruitment of research participants as community research associates were highly familiar with fellow community members, and resulted in an overall higher quality of research results and outcomes. Southern-based team members co-developed community research associate training workshops by collaborating with Ikaarvik:Barriers to Bridges, which is an Arctic Inspiration Prize winning “program that works with Arctic youth to be the bridge between research and their communities” (https://ocean.org/our-work/arctic-connections/ikaarvik-barriers-to-bridges/). Training workshops were planned via email, phone calls, and in-person meetings but were iteratively improved upon after each community case study based on feedback and observations. Community mapping workshops were also pre-planned prior to arriving in communities and involved reviewing relevant digital and hard copy participatory mapping methods, pilot testing with project partners, and map scaling and preparation.

#### Map scaling and preparation

In preparation for the training and community mapping workshops the southern-based team members communicated with each community that was participating in the research via email, phone calls, and in-person meetings when possible to identify the geographic region of interest for their community. This was an important step as it ensured that the maps used for the workshops reflected the relevant region used by community members for hunting and other cultural activities. In some communities, the geographic range from the main community was relatively small whereas for others the range was very large. Maps were purchased at an appropriate scale for each community based on their suggested use ranges so that the maps would enable participatory mapping at a useful size. These base maps were overlaid with multiple sheets of clear plastic which enabled participants to mark-up certain areas by season or theme and to avoid a single map becoming too ‘messy’. The project team used construction-grade vapour barrier purchased at a local hardware store and cut sheets to reflect the size of the maps used for each community. A poster-sized print-out of the portion of the Low Impact Shipping Corridors located within the study area was also prepared for mapping and workshop discussions (data provided by Canadian Hydrographic Service via a Memorandum of Understanding). To enable digitization of features mapped by participants during the workshops, a base map of Arctic Canada showing only coastlines was created using ArcGIS^Ⓡ^ software by Esri using topographic data from Natural Resources Canada. The paper maps used Universal Transverse Mercator projection, and the finished maps utilized Lambert Azimuthal Equal Area projection.

### Identification of community research associates

Before arriving in each community, our community partners who had been identified during the selection of community case studies identified four to ten local youth (aged 15 to 45 years of age ideally fluent in both English and Inuktut i.e., the Inuktitut or Inuinnaqtun dialect most common in that Inuit community) with an interest in research, to participate in the ACNV training workshop. Youth included high school students, delegates from local organizations, and graduates of relevant college programs. Many were active hunters or guides, or active on the land through programs such as Young Hunters and Junior Canadian Rangers. Having prior research experience was beneficial but not mandatory. Community research associates were paid (CAD25-40/USD19-30 per hour) (rates varied based on recommendations made by community partners) to attend a three-day long co-learning and participatory mapping training workshop. Many of the youth were then hired, at the same rate, to co-facilitate community mapping workshops and interviews that took place immediately following the training workshop. These youth were also hired to assist with results sharing and verification exercises that took place after results were digitized and analysed.

### Ethics procedures and research licensing

Once the project plan, methods, and community research associate training approaches were established, it was necessary to obtain a number of research and Indigenous ethics licenses considering the project involved human participants and the documentation of traditional and Inuit knowledge. Ethics approval was secured from the University of Ottawa (uOttawa) Research Ethics Board in early 2016 and was used as a template to obtain research ethics licenses from Aurora Research Institute and Nunavut Research Institute that were required in order to conduct research in Inuit Nunangat. Application information was provided in both English and Inuktut and also included information on protocols for; (1) data collection, (2) participant selection, (3) short and long-term use of data, (4) documenting and securing Inuit knowledge, (5) ensuring regional and local benefits, and (6) communicating results. To obtain Aurora Research Institute and Nunavut Research Institute research licenses it is vital to have the support of local community organizations and leaders, and the names and contact details of local project partners that the team had established were also provided.

## In the communities

### Co-learning and training workshops with community research associates

Southern-based team members travelled to communities approximately 5 days prior to the anticipated co-learning and mapping training workshop start date. They met with community partner organizations informally (e.g., Village Office Liaison, HTA manager) and formally (e.g., as a scheduled delegate at Hamlet Council, HTA Board, Community Corporation meetings), purchased refreshments, and set up all training materials in a dedicated (rented) space in the community (e.g., Hamlet chambers, church hall, classroom). At each three-day long co-learning and mapping training workshop southern-based team members, community partners and community research associates learned from each other. Discussions involved theoretical concepts and practical applications of research methods. Discussion topics included:

(1) Why this group of young people was selected to work together with this team of southern-based researchers, and the reason for gathering;
(2) Defining research and community-based research, the importance of conducting (locally-relevant) research, northern-based and southern-based researchers, comparing qualitative and quantitative research, links between scientific knowledge and Inuit knowledge, and the benefits of using both when conducting research;
(3) Defining shipping, why involving communities in shipping-related research was important, how to conduct community-based research and how to conduct mapping about shipping and local marine use areas;
(4) Refining questions to be asked at community mapping workshops and interviews, as well as question translation, back-translation, mapping methods, note-taking, co-facilitation techniques, research ethics protocols, research participant inclusion criteria, interpreter and research participant selection and recruitment; raising community awareness about the project; and
(5) Pre-test (practice run) followed by further refinement of mapping workshop methods and questions, and selection of community research associate roles during data collection (e.g. co-facilitator, note-taker etc.).

Community research associates were invited to complete a photo release form indicating their preference around anonymity or credit in project outputs, and were invited to have their photo taken for inclusion on the ACNV project website team member page (http://www.arcticcorridors.ca/about/). Parents and guardians of those under the age of 18 indicated their permission by completing the form.

#### Community mapping workshop question refinement

During the training workshop, a southern-based team member read draft questions for the community mapping workshop aloud, one at a time, to the community research associates who assessed question appropriateness. This included ensuring that questions were easily understood, non-technical, and relevant to the community. Question phrasing was revised accordingly. With the assistance of a skilled interpreter, community research associates translated and wrote down each question in Inuktut. Prompted by the southern-based team member they translated each question back into English to confirm that the original question nuance was maintained and that the questions were not leading once translated. When nuances misaligned the Inuktut and English question phrasing were refined iteratively. An example is the scientific term *impact* which can be expressed in Inuktut using words more similar to *touch* or *hit*. The latter two may lead participants’ answers to differ from the former. This process was time-consuming. Frequent breaks were required.

#### Mapping methods and note-taking training

Southern-based team members trained community research associates in mapping and note-taking methods through oral presentations which included showing example final products (e.g., digitized maps, the ACNV website and community reports), hands-on demonstrations, and activities (e.g., working in interviewer/interviewee pairs to map predetermined locations and travel routes within their hamlet such as specific homes, schools, and stores on a Googlemap printout of the community). The mapping exercises were guided by the conventions outlined in Tobias [17]; particularly Chapter 12 entitled ‘Recording spatial data - marking features on maps’. However, mapping conventions were tailored to suit ACNV project needs and applications, as outlined below. As Tobias [17] suggests, geographic information system analysts were involved in developing the mapping methods. Community research associates were trained to apply a specific series of mapping conventions that they employed during community mapping (data collection) workshops (see section on community mapping workshops and interviews for details).

Using English-Inuktut interpreters, the southern-based team members and community research associates practiced co-facilitating the workshop by asking discussion questions and encouraging participants to share their knowledge, and conducted mapping and note-taking using the methods they were just trained in. This was done during a half-day pre-test (or practice run) exercise conducted with 1 to 2 community members who met the inclusion criteria. Immediately following the pre-test, community research associates were asked to reflect on their experiences in their various roles and together the group problem-solved and strategized techniques to improve upon during the upcoming community mapping workshop. Each community research associate played a different role during the community mapping workshop (e.g., asking certain questions, taking notes, photographing participants, general assistance, or observing - an unpaid position) and these roles were determined ahead of time. Finally, the full group spent some time iteratively refining the research questions as was considered necessary.

### Co-identification and recruitment of mapping workshop participants

With the southern-based team members, community research associates raised community awareness about the ACNV research project, shared their role in the project, and recruited potential research participants. They did this at public events and in high-traffic locations (e.g., community meetings, flea markets, a table at the grocery store entrance) as well as on local radio at known high-listener times, and through Facebook posts in local group pages, many of which were ‘closed’ groups thus were not accessible to southern-based team members. Southern-based team members and community research associates had conversations with interested community members to further explain the project goals and to dispel any possible misunderstandings about the research topic that arose. Due to historic colonization and unethical research practices in the past that occurred in Arctic Canada this step in the research process was very important.

Inclusion criteria for community mapping workshop (research) participants were initially developed by southern-based team members but were refined based on recommendations from community partners and research associates. For example, initially Elders that were not active on the land or marine environment were excluded but based on local recommendations were then re-included and, in the end, proved to be important contributors and mentors. Southern-based team members and community research associates met in person with local organizations (e.g., Hamlet Council, HTA Board) in order to identify potential research participants who met agreed upon criteria such as being an Elder, or being currently active on the land; having knowledge of a variety of geographical locations; and including a range of individuals based on age, gender and local organization affiliation.

Working individually or in pairs according to personal preference, each community research associate recruited 1 to 2 participants through methods identified by community partners and research associates as suitable within their community (while respecting research ethics and licensing protocols). These included initial introductory phone calls, in-person visits at work or home, and hand-delivery of invitation letters in one or both relevant languages (English or Inuktut).

### Community mapping workshops and interviews

Community mapping workshops were held for two days in each case study community with the objectives to record and map culturally significant marine use areas, perspectives on the impacts of shipping, and recommendations for marine vessels transiting the Low Impact Shipping Corridors. Workshops were co-facilitated by southern-based team members and community research associates in English and Inuktut, simultaneously interpreted by skilled, hired interpreters, and audio-recorded. Light refreshments were provided, participants went home for lunch as per local preferences, and each was given an honorarium in appreciation (CAD200-300/USD152-228 at the time of submission per day) according to local practices.

Each workshop was opened with a prayer led by a local Elder and brief introductions, followed by a presentation by the southern-based research team including autobiographical details and photos (e.g., hobbies, family), information about the research project, funders, supporters, marine vessel traffic trends, and the Low Impact Shipping Corridors, and informed consent. Research participants were asked if they consented to being audio-recorded, photographed, and having notes taken. Southern-based team members orally reviewed the contents of the invitation letter and consent form, received oral consent from participants, and invited participants to complete hard copy consent forms indicating their preference around anonymity or credit at the end of the community mapping workshop. Options included anonymity, or being credited by name and/or brief biography and/or photo in project outputs. It was important to offer to give credit where credit was due. In addition, highlighting and publicizing workshop participants’ identity enabled community members who were not involved in the project to better evaluate the verity of project outputs i.e., local (and other) audiences could assess the likelihood of accuracy of the report and maps based on their perception of workshop participants’ expertise.

Mapping exercises and group discussions were conducted using a specific set of methods that community research associates were trained in. The approach and methods included the following:

1. The base map was taped to the table and marked with one cross approximately 1 cm long in each quadrant of the map. The base map was overlaid with clear plastic. The plastic overlay was taped to the table and marked with the same four crosses as on the base map (to enable accurate future re-fitting of the plastic to the map).
2. Each plastic overlay was labelled with the date, community name, and season or topic.
3. Following the question guide, community research associates asked research participants to point to or trace with their finger the location of a feature. Community research associates then drew the feature on the plastic overlay, using a short line (called a leader) to connect the feature to a number. Each number was a unique identifier, starting with number 1 and ascending consecutively throughout the entire workshop and across all overlays. Contrary to Tobias [17] predetermined codes were not used because participant remarks and features could not be predicted i.e., codes could not be generated prior to the community mapping workshop.
4. In dedicated notebooks, community research associates recorded the plastic overlay label (see number 2 above), and each unique identifying number as it was marked on the overlay, along with a brief description of each feature, e.g., ‘1 - seal harvesting area’. Unlike Tobias [17] notes were documented in notebooks and not on overlay margins, for reasons of clarity and neatness. Note-takers summarized research participant perspectives in point form in English to ensure key comments were documented. The identity of each person providing the data was not coded, in an effort to protect anonymity.
5. While a variety of colours of fine-tipped Sharpie-type markers were used, colour was not attributed any meaning, rather it was used only to enable clear differentiation between unique features (lines, points and polygons/shapes; [17], pg. 233–234). The same colour was used for each feature-leader-number combination to ensure clarity particularly for overlapping features. Unlike Tobias [[17], pg. 235] colour-category runs were not used, as this approach seemed unnecessarily complicated.
6. As suggested in Tobias [17] polygons were completely closed, errors were corrected using diagonal cross-hatching (pg. 233) and “discrete networks of joined line segments [were] coded as separate features” (pg. 249). As many overlays as were needed to maintain clarity were used, and “features were coded enough times to prevent both ambiguity and unnecessary clutter” (ibid, pg. 240).
7. In this study, pairs of leaders frequently crossed particularly when marking local travel routes (ibid. pg. 236), a practice Tobias [17] cautions against. Textures were not used to differentiate between travel routes (ibid, pg. 250) rather travel routes were mapped according to season and type using a solid line. Travel routes were not bracketed at either end as confusion arose in distinguishing brackets from leaders due to the density of the travel routes (i.e., digitizers could not tell where the bracket ended and the feature label began).
8. Although recommended by Tobias [17] direction of travel was not indicated with arrows (pg. 254) as community members reported following the same travel routes regardless of direction of travel. The exception was animal migratory routes as the direction of animal travel changed seasonally.
9. To ensure interviewer bias was prevented, community research associates ensured that “the extent of the polygon [was] based on the respondent's information and not the interviewer's assumption” (ibid, pg. 241). For instance, if a participant said “I collect eggs on such-and-such island”, rather than tracing the perimeter of that island, the community research associate replied, “Please show me where on the island you collect eggs” and the participant would indicate the appropriate location. Only then would the community research associate draw the feature on the overlay.
10. Plastic overlays were replaced when they became crowded (i.e., features were at risk of being difficult to distinguish) or when map foci changed significantly (e.g., new season). As each plastic overlay was completed a southern-based team member took a high-resolution photo from directly above i.e., held their camera over the map. Then the completed plastic overlay was removed for safe storage. This process, numbers 1 through 10, was repeated until mapping concluded i.e., all questions were answered to participants’ satisfaction.

Near the end of the workshop, southern-based researchers facilitated a discussion on next steps including timelines for results verification and data sharing events and protocols for data management and sharing. The workshop closed following statements of appreciation by southern-based team members, community research associates, community partners, and research participants.

Supplementary, semi-structured key informant interviews were conducted when identified potential participants were unable to attend the two-day long workshop (e.g., full-time employment, lack of daytime childcare, lack of transportation, restricted mobility/housebound) or were otherwise occupied during the workshop (e.g., interpreting, co-facilitating). Interviews were conducted in the location of interviewees’ choice (e.g., home, office, public meeting space), in English and Inuktut, simultaneously interpreted and audio-recorded. Interviews lasted 1.5 h on average.

### Digitizing community mapping results

Trained undergraduate, graduate, and postdoctoral data analysts (research assistants) conducted all aspects of digitization of community mapping results; four of whom travelled to Arctic Canada and participated in training and/community mapping workshops and/or results verification exercises. All data management procedures described below were planned by data analysts in consultation with the uOttawa Geographic, Statistical and Government Information Centre, before digitizing commenced. All stages of the workflow and procedures were documented so that the digitization process and storage of data were consistent across communities and data analysts.

Initial data analyses involved the creation of draft community reports that included preliminary digitized maps showing participants’ combined knowledge from the workshops. For 11 communities, report writing and digitization were conducted at the uOttawa, Environment Society, and Policy Group (ESPG) laboratory (see www.espg.ca) and a southern-based team member returned to the community months later to conduct results verification exercises. For three communities, due to budgetary (e.g., flight cost of 8000CAD/6900USD per person) and time (fiscal deadlines) restrictions, a data analyst was included in the team of southern-based researchers and this person travelled to communities to co-facilitate training and community mapping workshops, then remained in the community to conduct report writing and digitization so that verification exercises could take place during the same trip. In those communities results verification exercises were conducted within one week of the community mapping workshop.

**Fig. 2:**
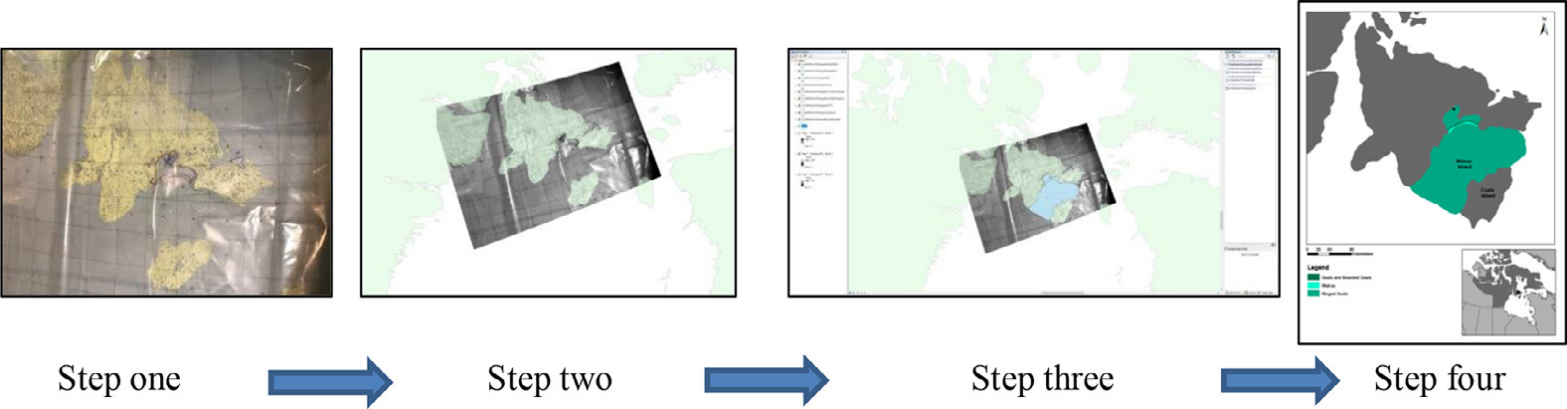
Digitization in ArcGIS from a photograph of a printed map and overlay with hand-drawn features to a digital map.

The following four steps shown in Fig. 2 were utilized to convert the hard copy workshop maps to digital maps.

Step one: Photographs were taken of the paper maps and overlays with the hand-drawn features marked during the community mapping workshops, and imported into ArcMap (Esri ArcGIS^Ⓡ^ software).

Step two: Using the georeferencing toolbar, the imported image was assigned real-world coordinates using anchor control points to the digital base map; thus, converting the image to a spatially referenced map.

Step three: Map features were digitized using the editor tool in ArcMap. To do this, shapefiles were created by tracing each feature in the georeferenced photograph of the map. Each shapefile represented a geographical feature or features as identified by community members. These shapefiles were grouped into three different categories (human activities, wildlife activities, or significant marine features) and relevant attributes were assigned (e.g., community, season, location, category/group, type/species). The same process was applied for the recommendations for the Low Impact Shipping Corridors with relevant attributes recorded (e.g., community, type, details such as “no anchoring”, or “preferred corridor”).

Step four: Maps (figures) were created that included a legend, graticule, scale bar, and north arrow, and all shapefiles showing: (a) human activities and features of cultural significance according to season, (b) wildlife activities according to season, (c) significant marine features according to season, and (d) recommendations regarding the Low Impact Shipping Corridors.

There were instances where shapefiles showing different features in the same season overlapped with each other (e.g., beluga whales and walruses were found in the same areas). When this occurred, a sufficient number of maps were created to ensure that all features were clearly visible.

#### Results verification and data sharing events in communities

An English version summary report of key findings was drafted by the research team and was presented to research participants during verification events in order to ensure that maps were digitized accurately and that information was summarized and interpreted correctly. These reports and associated maps were verified with community research associates and other partners during in-person meetings. After this first step, southern-based researchers and community research associates co-facilitated in-person verification with available research participants. Reports were reviewed line-by-line and hardcopy maps were displayed. Participants were encouraged to comment on and annotate the draft documents and maps wherever discrepancies were noted. Formal verification exercises were conducted in English and Inuktut, simultaneously interpreted, and all comments were recorded. In addition, a series of informal community meetings were held in public spaces commonly used for that purpose (e.g., church or community hall) to allow other community members to view project results and to provide further input on the validity of the maps and findings. Hard copies of maps were displayed for the public to comment on. These events were advertised in English and Inuktut by community research associates and partners on local radio and Facebook pages and using hard copy posters in high traffic areas (e.g., Hamlet office, grocery store). Light refreshments and door prizes were provided during the events. The format often involved a brief joint presentation by community research associates and partners, research participants, and southern-based team members followed by a question and answer period.

## After in-community research activities

### Post-verification digitization edits

#### Mapping

Participant verification feedback that was collected during the results verification and sharing events (described above) was incorporated into the final versions of the community maps. This involved correction of shapefile features, adding new features and adding or changing attributes in ArcMap (as explained in Step 3 above). The final attribute data assigned to each shapefile were exported as text files and opened in Microsoft Excel software and saved as Excel spreadsheets. The final versions of all shapefiles were saved in a post-verification folder. This maintained strict version control, to ensure that only post-verification results were shared and used in publications and reports.

### Transcription and textual analysis

#### Workshop and interview data analysis

To analyse the data focused on community-identified recommendations for Low Impact Shipping Corridors published in [4], all of the community mapping workshop and interview audio recordings were transcribed verbatim in English. These transcriptions were analysed using conventional content analysis [12]. Firstly, researchers familiarized themselves with the data by reading through the transcripts, community reports, and also by listening to the audio recordings. Following this, coding was conducted using NVivo software (QSR International (Americans) Inc. 35 Corporate Drive, Burlington, MA). Some overarching codes regarding recommendations for the Low Impact Shipping Corridors were created interactively as community research was completed. These codes were:

- Area to avoid
- Preferred corridor (new community recommended corridor to replace government proposed corridor)
- No ice breaking
- Modify vessel operation
- Speed
- Noise
- Restricted shipping seasonally

Additional codes were identified during the analysis. Ultimately, over 200 codes were created during the first coding process, these included overarching themes such as impacts of shipping, climate change, security, and recommendations. Following this, categories and themes were created and common codes were placed within these overarching categories. While we used the above codes as data for [4], it also enabled the researchers to organize and tag the entire dataset thus facilitating additional outputs and impacts. This was also important as there was often additional information that provided useful context and understanding for the overall analysis.

### Integration of spatial and non-spatial data

Non-spatial and spatial data were cross-referenced to ensure validity and to provide the context needed to explain why certain recommendations were suggested by research participants. For example, whenever a recommendation was made by a community that was referenced spatially on the map, the reasoning and discussion behind it (that were recorded, transcribed and coded) were then linked using a specific reference code to that spatial recommendation. This enabled access to the full context of why and how the recommendation was made and the discussions surrounding it. This was part of the data triangulation process and also enhanced research validity.

### Preparation and publication of community reports

#### Preparation

Participant verification feedback was incorporated into community reports. Select co-authors of each report (those present during community mapping workshops) copy edited reports to ensure that they were free of factual and grammatical errors as well as to improve fitness for purpose and readability. A graphic designer and select southern-based team members iteratively developed a single design template for the suite of 14 community reports. Copy (text) was provided to the designer as text files. Figures including maps were provided as high resolution (300 DPI at 7.5 inches) image files. Final community reports were produced in high and low (web-suitable) resolution and for print.

#### Publication

With the help of the uOttawa Scholarly Communication Librarian each report was assigned a unique digital object identifier (a combination of numbers, letters and symbols that permanently identify the report and document and link to it online). Reports were published in uOttawa's digital repository for research materials https://ruor.uottawa.ca. Reports were also published on a dedicated page of the ACNV project website http://www.arcticcorridors.ca/reports/.

### Data sharing and management

#### Data sharing with partners

Electronic versions of community reports were emailed to community research associates and community partners immediately upon publication (within 60 days post verification exercises). Professionally-printed spiral-bound (to enhance map-viewing ease) versions were mailed and/or delivered in-person to community partners for distribution and placement in strategic community locations (e.g., HTA office, Wildlife office, Hamlet office, high school). Using community Facebook groups, community partners and research associates as well as southern-based team members, posted links to the ACNV website reports page and notified community members where hard copies were locally available. The principal investigator annually e-mailed a one-page project progress update (in English, Inuktitut and Inuinnaqtun) to all community and other partners, stakeholders, and numerous media outlets outlining the prior year's achievements, results sharing exercises, and next steps.

In addition to conducting data sharing in each community (see *Results verification and data sharing events in communities*) the southern-based team members hosted a two-day long ACNV project results sharing workshop in Iqaluit, Nunavut, Canada (the territorial capital city and administrative hub). For the workshop discussion paper and workshop report see [5,6]. The 23 workshop participants included Government of Nunavut representatives from the departments of Environment and Economic Development and Transportation, including the Minister and Deputy Minister of Economic Development and Transportation, representatives from the Nunavut Planning Commission and Nunavut Tunngavik Incorporated, a community research associate, and academic researchers. Invitations for (travel) funded participation were extended to non-Iqaluit-based representatives of numerous territorial Institutions of Public Government, Government of Nunavut departments, and non-governmental organizations but due to scheduling and travel conflicts representatives were unable to attend.

Shapefiles of community-identified management recommendations for the Low Impact Shipping Corridors and community reports were shared with Government of Canada agencies directly involved in the prioritization of the Low Impact Shipping Corridors. In aggregate form, portions of the project results are publicly available at www.arcticcorridors.ca in written reports and maps for every community. In addition, a reflection and evaluation of the research methods and approach undertaken in the ACNV project has been co-authored by community partners, community research associates and southern-based research team members (see [1]). That manuscript provides unique insight into the benefits and challenges of engaging in community-based research and what partnered collaborative research means in practice. Readers are invited to consult these project outputs as additional resources.

#### Proprietary local knowledge returned to communities for ownership, stewardship, and archiving

Shapefiles of culturally significant marine use areas and community-identified management recommendations for the Low Impact Shipping Corridors were shared directly with community organizations, and Inuit corporations and organizations who retain ownership of the data. Tailored data ethics information briefs were acknowledged and signed by each data recipient. These included but were not limited to the following: (1) Proper acknowledgements should be made when using the data including to the communities, local researchers, and local knowledge holders that were involved in and that were leaders in the project and to the uOttawa ESPG research team; (2) The experts whose knowledge and perspectives are documented within this data, their communities, and representative bodies (i.e., Inuit organizations), all retain ownership, control and possession of this knowledge, and are guaranteed access to it; (3) The experts, the local communities, and representative bodies (i.e., Inuit organizations) retain ownership of the data as it is their cultural knowledge. Inuit, as a community and their representative bodies, own this information collectively in the same way that an individual owns his or her personal information. Interviews and focus group discussions are confidential; (4) The experts, their communities, and representative bodies (i.e., Inuit organizations) have access to this information regardless of where it is held. They also have the right to manage and make decisions regarding access to their collective information; (5) If at any time there is uncertainty about data ownership, the OCAP^Ⓡ^ regulations, the National Inuit Strategy on Research [10], and the ESPG at uOttawa should be consulted; and (6) All individuals working with the data must be made aware of the ethical requirements of using the data.

Data usage was also outlined in the data ethics information briefs. For instance, data were shared with the Government of Canada solely for the purpose of supporting decision-making for sustainable and respectful shipping in Inuit Nunangat and to support the prioritization of Low Impact Shipping Corridors. Data shared with one of the Inuit corporations were for the purpose of informing decision-making for marine protected areas, or other effective conservation-based measures within their jurisdictional region.

## Conclusion

In this article, the methods applied when conducting the ACNV project, and related observations, are shared. The methods presented here were utilized to (1) ensure scientific processes and outputs were robust and rigorous; and (2) to conduct research in a respectful, reciprocal manner i.e., through meaningful involvement of those affected by the research. Only through the collaborative efforts of a transdisciplinary team (rights holders, community members and organizations, government, transdisciplinary southern-based researchers, and non-governmental organizations) could this rigour be attained and this respect be afforded. These methods and observations are shared with the goal of informing future research initiatives, particularly transdisciplinary teams, including those involving southern-based (early career) researchers, working in Inuit Nunangat.

## Declaration of Competing Interest

The authors declare that they have no known competing financial interests or personal relationships that could have appeared to influence the work reported in this paper.

## References

[1] Carter N.A., Dawson J., Simonee N., Tagalik S., Ljubicic G. (2019). Lessons learned through research partnership and capacity enhancement in Inuit Nunangat (the Inuit homeland in Arctic Canada). Arct.

[2] Castleden H., Garvin T., Nation Huu-ay-aht First (2008). Modifying photovoice for community-based participatory Indigenous research. Soc.Sci. and Med..

[3] Chénier R., Abado L., Sabourin O., Tardif L. (2017). Northern marine transportation corridors: creation and analysis of northern marine traffic routes in Canadian waters. Trans. GIS.

[4] Dawson J., Carter N., van Luijk N. (2020). Infusing Inuit and local knowledge into the low impact shipping corridors: an adaptation to increased shipping activity and climate change in Arctic Canada. Environ. Sci. Policy.

[5] Dawson J., Carter N.A., Reid M.B. (2019). Development and *Management* of Low-Impact S*hipping* Corridors in Nunavut: *Workshop Discussion Pape*r. http://www.arcticcorridors.ca/?acr_download=%2Fwp-content%2Fuploads%2F2020%2F01%2F2019-Workshop-Discussion_Paper.pdf.

[6] Dawson J., Carter N., Reid M. (2019). Development and Management of Low-Impact S*hipping* Corridors in Nunavut: a *Workshop Repor*t. http://www.arcticcorridors.ca/wp-content/uploads/2019/11/ACNV_Nunavut_Workshop_Report_Final.pdf.

[7] Dawson J., Pizzolato L., Howell S.E.L., Copland L., Johnston M.E. (2018). Temporal and spatial patterns of ship traffic in the Canadian Arctic from 1990 to 2015. Arct.

[8] Dawson J., Porta L., Okuribido-Malcolm S., deHann M., Mussells O. (2016). Proceedings of the Northern Marine Transportation Corridors Workshop.

[9] International Fund for Agricultural Development (IFAD). (2009). Good practices in participatory mapping. http://www.iapad.org/wp-content/uploads/2015/07/ifad_good_practice_in-participatory_mapping.pdf.

[10] Inuit Tapiriit Kanatami. (2019). National Inuit Strategy on Research. https://www.itk.ca/wp-content/uploads/2018/03/National-Inuit-Strategy-on-Research.pdf.

[11] Kue J., Thorburn S., Keon K.L. (2015). Research challenges and lessons learned from conducting community-based research with the Hmong community. Health Promot. Pract..

[12] Nowell L.S., Norris J.M., White D.E., Moules N.J. (2017). Thematic analysis: striving to meet the trustworthiness criteria. Int. J. of Qual. Methods.

[13] OPM (Office of the Prime Minister). 2016a. United States-Canada joint Arctic leader's statement. https://pm.gc.ca/eng/news/2016/12/20/united-states-canada-joint-arctic-leaders-statement.

[14] OPM (Office of the Prime Minister). 2016b. Canada's oceans protection plan: creating stronger indigenous partnerships and engaging coastal communities. https://pm.gc.ca/eng/news/2016/11/07/canadas-oceans-protection-plan-creating-stronger-indigenous-partnerships-and.

[15] Pew Charitable Trusts (2016). The *Integrated* Arctic c*orridors* framework: Planning f*or Responsible Shipping* in Canada's A*rctic Water*s. https://www.pewtrusts.org/~/media/assets/2016/04/the-integrated-arctic-corridors-framework.pdf.

[16] Pizzolato L., Howell S.E.L., Dawson J., Laliberté F., Copland L. (2016). The influence of declining sea ice on shipping activity in the Canadian Arctic. Geophys. Res. Letters.

[17] Tobias T. (2009). Living proof: The essential Data-Collection Guide For Indigenous use-And-Occupancy Map Surveys.

